# Toxicologic Concerns with Current Medical Nanoparticles

**DOI:** 10.3390/ijms23147597

**Published:** 2022-07-08

**Authors:** Tsai-Mu Cheng, Hsiu-Yi Chu, Haw-Ming Huang, Zi-Lin Li, Chiang-Ying Chen, Ya-Jung Shih, Jacqueline Whang-Peng, R. Holland Cheng, Ju-Ku Mo, Hung-Yun Lin, Kuan Wang

**Affiliations:** 1 Graduate Institute for Translational Medicine, College of Medical Science and Technology, Taipei Medical University, Taipei 11031, Taiwan; tmcheng@tmu.edu.tw (T.-M.C.); chuxiuyi@tmu.edu.tw (H.-Y.C.); 2 Taipei Heart Institute, Taipei Medical University, Taipei 11031, Taiwan; 3 School of Dentistry, College of Oral Medicine, Taipei Medical University, Taipei 11031, Taiwan; hhm@tmu.edu.tw; 4 Graduate Institute of Nanomedicine and Medical Engineering, College of Medical Engineering, Taipei Medical University, Taipei 11031, Taiwan; lizilin919@tmu.edu.tw (Z.-L.L.); janechen0215@tmu.edu.tw (C.-Y.C.); shihyj@tmu.edu.tw (Y.-J.S.); 5 Graduate Institute of Cancer Biology and Drug Discovery, College of Medical Science and Technology, Taipei Medical University, Taipei 11031, Taiwan; tracy451210@gmail.com; 6 Cancer Center, Wan Fang Hospital, Taipei Medical University, Taipei 11031, Taiwan; jqwpeng@nhri.org.tw; 7 Department of Molecular & Cellular Biology, University of California, Davis, CA 95616, USA; rhch@ucdavis.edu; 8 TMU Research Center of Cancer Translational Medicine, Taipei Medical University, Taipei 11031, Taiwan; 9 Traditional Herbal Medicine Research Center of Taipei Medical University Hospital, Taipei Medical University, Taipei 11031, Taiwan; 10 Pharmaceutical Research Institute, Albany College of Pharmacy and Health Sciences, Albany, NY 12208, USA

**Keywords:** nanoparticles, medical applications, nanotoxicity, cytotoxicity, inhalation, ingestion

## Abstract

Nanotechnology is one of the scientific advances in technology. Nanoparticles (NPs) are small materials ranging from 1 to 100 nm. When the shape of the supplied nanoparticles changes, the physiological response of the cells can be very different. Several characteristics of NPs such as the composition, surface chemistry, surface charge, and shape are also important parameters affecting the toxicity of nanomaterials. This review covered specific topics that address the effects of NPs on nanomedicine. Furthermore, mechanisms of different types of nanomaterial-induced cytotoxicities were described. The distributions of different NPs in organs and their adverse effects were also emphasized. This review provides insight into the scientific community interested in nano(bio)technology, nanomedicine, and nanotoxicology. The content may also be of interest to a broad range of scientists.

## 1. Background

The highly expanding field in nanotechnologies, from “smart drug” packaging that can reach the central nervous system and accurately target tumor cells [1], to self-cleaning glass, from nano-gold embedded in odorous socks to the development of stealth fighter jets, artificial muscle to desalination plants, from safer nuclear energy to better clinical diagnosis, there are few areas of human effort that do not believe that nanotechnology can play an important role. The pace of development of nanotechnology is amazing. Nanotechnology’s continued progress has led to the development of nanoscale therapies to alleviate many complex diseases. This has brought to market several new nanomaterials and their composites [2], including liposomes, polymer nanoparticles (NPs), dendrimers, and nanostructured lipid carriers. NPs efficiently cross the membrane barrier, are distributed throughout the body by translocation into the bloodstream, and exhibit their role in organs and tissues at the cellular and molecular levels. The interaction of NPs with cells can cause nanotoxicity [3,4,5]. The use of nanomaterials can be commonplace. However, those applications are still determined by the high reward–risk ratio. As nanotechnology progresses, it becomes more concerned about the impact and potential impacts of exposure of ecosystems to these substances. There are few studies on assessing the toxicity of NPs in biological systems. Therefore, with limited knowledge of the toxicity profile of monotherapy, side effects are ignored. However, nanotoxicology has recently evolved into an attractive research category. NPs are very small in size, have a large surface-area-to-volume ratio, and have a large shape and surface function. The narrow particle size distribution, large surface-to-mass ratio, and surface properties of NPs are highly associated with nanotoxicity, which can cause damage at the cellular, intracellular, molecular, and protein levels. For instance, exposure to surrounding aerosols cannot be predicted to accelerate the thickening of the carotid artery wall [6]. However, we know that this happens in human epidemiological studies [7]. Instead of interdisciplinary applications of NPs, research regarding toxicity issues and the impact of these substances on public health and the environment is still in its early stages [8]. Therefore, it is very important to investigate the mechanism of nanotoxicology. Toxicity assessment needs to be an integral part of the development of nanotherapy using a variety of toxicity assessment models. The purpose of this review was to investigate the various nanostructures of therapeutic delivery systems and their physicochemical properties that adversely affect human biology [9]. In addition, this review aimed to provide a wide range of information on routes of NPs entry into living organisms, their organ distribution, their mechanism of action, and their potential impacts on human health.

## 2. Sources of Nanoparticles

**Figure 1:**
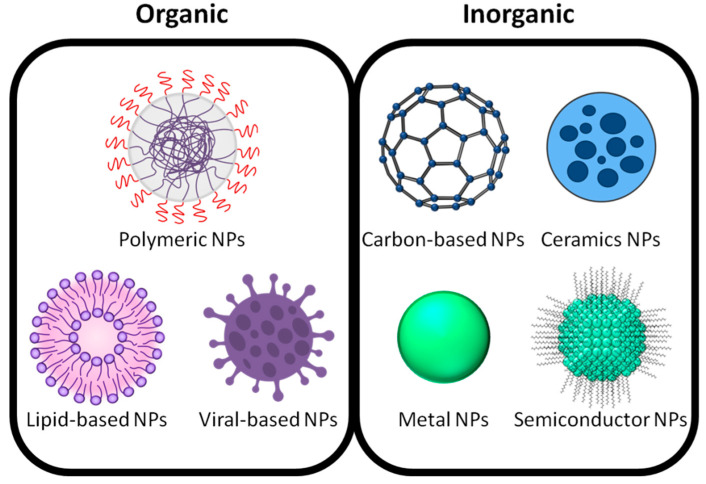
Different types of NPs. Organic NPs: polymeric NPs, lipid-based NPs, and viral-based NPs; inorganic NPs: carbon-based NPs, metallic NPs, ceramic NPs, and semiconductor NPs.

NPs can be classified into different classes based on their characteristics, shape, or size (Table 1). The different groups include fullerenes, metallic NPs, ceramic NPs, polymeric NPs, lipid-based NPs, and viral-based NPs (Figure 1) [9,10,11]. Among them, carbon nanotubes, metallic NPs, lipid-based NPs, and viral-based NPs have emerged as powerful tools in medical applications [12,13,14,15,16]. On the other hand, metallic NPs such as gold nanoparticles (AuNPs) are applied to a wide range of medical applications, including drug and gene delivery, photothermal therapy, photodynamic therapy and radiation therapy, diagnosis, X-ray imaging, computed tomography, and other biological activities [17].

The size, hydrophobicity, and charge of NPs determine the physical and chemical properties of NPs and play important roles in their metabolism, absorption, distribution, and excretion [18,19]. The NP size is an important parameter that determines the pharmacokinetics of NPs [20]. Their optical properties have been reported to be size-dependent, giving them different colors due to absorption in the visible region [21]. Due to their large surface area and nanoscale size, NPs have unique physical and chemical properties [18,22]. Their reactivity, toughness, and other properties also depend on their unique size, shape, and structure [9]. These properties make them suitable candidates for a variety of commercial and domestic applications, including catalysis, imaging, medical applications, environmental applications, and energy-based research. The size of NPs also regulates their transportation into cells and interaction with the immune system [23].

**Table 1:** Types of nanoparticles and their medical applications.

Type	Formation and Compositions	Applications	References
Carbon-based NPs	Fullerenes Carbon nanotubes (CNTs)	Carbon nanotubes are used widely in biomedical applications because of their multipurpose properties. They have been applied for carrying anticancer drugs or genes and proteins for chemotherapy.	[24,25,26,27]
Metal NPs	Alkali and noble metals such as Cu, Ag, and Au.	Noble metal-based NPs are applied in medical fields that needed high biocompatibility, stability, and large-scale production with the possibility of avoiding organic solvents.	[28,29,30,31]
Ceramics NPs	They are amorphous, polycrystalline, dense, porous, or hollow forms.	Medical technologies use nanoceramics for bone repair. In addition, they have been used in catalysis, photocatalysis, photodegradation of dyes, and imaging applications.	[32]
Semiconductor NPs	They possess properties between metals and nonmetals. Semiconductor NPs used in biosensing generally contain metals with nonmetallic elements.	Photocatalysis, photo optics, and electronic devices.	[33]
Polymeric NPs	They normally are organic-based nanospheres or nanocapsules primarily. The former are matrix particles that are generally solid, and other molecules are adsorbed to the outer boundary of the sphere. Nanocapsules are completely encapsulated mass particles.	Polymers with superior biocompatibility do not induce immune reactions or stimulate inflammation in contact with the human body. The advantages of synthetic polymers are their stability, excellent mechanical properties, and degradability. Polymers are biocompatible, biodegradable, non-toxic, and popular in medical applications such as drug delivery, wound plug dressings, stents, and tissue engineering.	[34,35,36,37,38]
Lipid-based NPs	Lipid-based NPs classified as lipid moieties including liposomes, solid lipid nanoparticles (SLN), and nanostructured lipid carriers (NLC).	Lipid-based NPs are used effectively in biomedical applications. They are used for various applications such as drug carriers, delivery, and RNA release in cancer therapy and COVID-19 vaccines.	[16,39,40]
Viral-NPs	Genetically engineered VNPs and chemically engineered VNPs.	Viral NPs serve as multipurpose tools for medical applications. Genetically engineered VNPs are used as vaccines. Chemically engineered VNPs are for targeted drug delivery and biomedical imaging.	[11,41]

The surface properties of NPs determine their hydrophilicity or hydrophobicity, and a variety of biological responses as well. These biological activities include cell uptake, interaction with plasma proteins, particle removal, and immune responses [42]. Furthermore, the physical surface properties of metal NPs such as silver, gold, and iron oxide NPs can be modified to act as drug carriers via d-mode active management [43]. However, the use of organic nanotransporters is considered preferable because the physical and chemical properties can be fine-tuned by changing the chemical composition, shape, size, structural morphology, and surface properties [44]. The nano-delivery efficiency of natural remedies also depends on their molecular weight. Increased molecular weight generally leads to reduced delivery efficiency of charged compounds, which in turn leads to their reduced bioavailability [45]. Hyaluronic acid (HA) is a biopolymer based on natural polysaccharides. It shows biocompatibility and non-toxicity properties; therefore, it is frequently used as a biomaterial for controlled drug release [46,47]. Hybrid hyaluronan-superparamagnetic iron oxide NPs targeted highly against and showed cytotoxicity glioblastoma U87MG cells [48]. Various nanotherapeutic delivery systems can provide different health benefits, depending on their properties, carrier properties, and desired therapeutic use [49,50].

## 3. Routes of Nanoparticle Uptake to the Human Body

We are all constantly exposed to NPs in the surrounding aerosols. The extrinsic ingestion of artificial NPs results primarily from hand-to-mouth contact at work between workers, engineers, and scientists working on cutting-edge products in the laboratory. NPs such as AuNPs can be exposed during either development or synthesis. It also cannot exclude routes such as dermal absorption inhalation, ingestion from implants, airborne adherence, surface materials, and results of AuNP-composite attached to consumer products in homes, markets, waste disposal, and other outdoor places [51,52,53,54,55]. In addition, NPs can be ingested directly via food, drinking water, drugs, or drug delivery systems. However, they can be up-taken during applications through direct ingestion or injection into our bodies or waste disposal [54,55]. The effects of the up-taken NPs are uncertain. However, related studies conducted with volunteers in this area are limited. The interesting study by Kuschner et al. indicated that exposure to fine and ultrafine magnesium oxide particles produces no evidence of lung inflammation [56]. This is a potentially important discovery as it casts doubt on the theory that the physical properties of a particle dominate the response and shows that the chemistry of the particle is important. This study does not appear to be repeated or followed up. Inhaled ultrafine particles are so small in size that they can settle in the olfactory mucosa and migrate to the central nervous system (CNS), causing neurotoxicity. CNS can be an important target for exposure by inhalation or intranasal injection of NPs. Exposure to NPs is associated with a range of acute and chronic effects ranging from exacerbations of inflammation, asthma, and metal fume fever to fibrosis, chronic inflammatory lung disease, and carcinogenesis. Various studies have shown that inhaled or infused NPs can enter the systemic circulation and migrate to different organs and tissues.

## 4. Therapeutic Nano-Delivery Systems

Most types of cancer are known as heterogeneous conditions [57]. Genomic modification plays an important role in the development of tumors [58]. In addition, the complex signaling pathways involve the development of different types of cancer; the need to discover new methods of prevention and detection is crucial [59]. Chemotherapy and radiation therapy are traditional procedures to be used for different types of cancer. However, they are hampered by many side effects [60,61]. Due to the large number of adverse events observed with these therapies, it is imperative to continuously develop new and improved strategies for the management of cancer patients. Nanomedicines facilitate the creation of new methods for detecting chromosomal rearrangements and mutations for targeted chemotherapy.

Different types of NP vectors have been developed to deliver the drug to tumor sites, organs, and areas of interest [59,62,63]. Chemotherapy is a major therapeutic approach for the treatment of both localized and metastasized cancers. Anticancer drugs such as paclitaxel and docetaxel have exhibited poor solubility. Small molecule anticancer drugs for VEGFR inhibitors (e.g., cabozantinib and nintedanib) and compounds such as curcumin also exhibit similar concerns [64,65,66]. To avoid the biodegradation of the therapeutic agent and to extend its stability in the organism, several types of nanocarriers have been recently developed. Alternatively, gene therapies have been employed for cancer therapies via viral and non-viral vectors. The former have high transfection efficiency and have been applied for the treatment of prostate cancer, breast cancer [67], melanoma [68], and glioblastoma [69]. On the other hand, non-viral vectors are for safer consideration [70]. In addition, targeted therapy is another axis of the development of NPs [62].

There are several administration routes for anti-cancer drug delivery. Some of the NP formulations have offered improved and higher oral availability of low-water-soluble drugs. On the other hand, carbon nanotubes have an excellent ability to penetrate cell membranes and functionalize with almost any biomolecule, thus targeting and delivering drugs under appropriate environmental stimuli [71].

**Figure 2:**
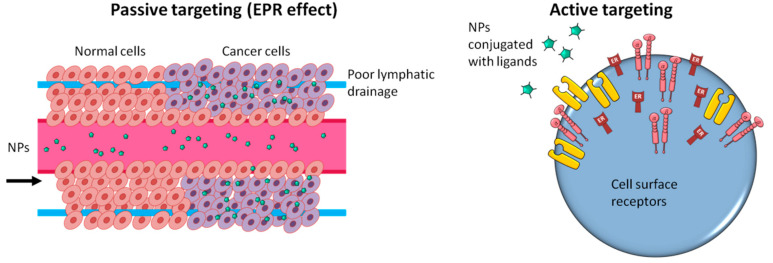
Medical NPs deliver via passive targeting or active targeting mechanisms. Two types of delivery systems are applied in drug delivery. One is a passive targeting system that does not differentiate between targeted cells and normal cells. The other is the active targeting system that delivers drugs specifically to the targeted cells based on cell surface receptors or biomarkers.

NPs can function through active or passive therapeutic targeting (Figure 2) [72]. After the passive nano-delivery mode, the charged therapeutic agent is released by corrosion or diffusion of the delivered nanocarrier. The active mode of administration allows the controlled release of the transported biomolecules to the target body part. In this mode of delivery, certain cell surface receptors or biomolecules are used as biomarkers to reach specific target sites [73]. By combining specific stimulus–response components, it is also possible to selectively target specific tissues or body parts, which may be triggered by specific stimuli, such as electric or magnetic fields, light, pH, heating, ultrasound, and contact with concentrated solutions, ionic solutions, or certain enzymes [74].

**Figure 3:**
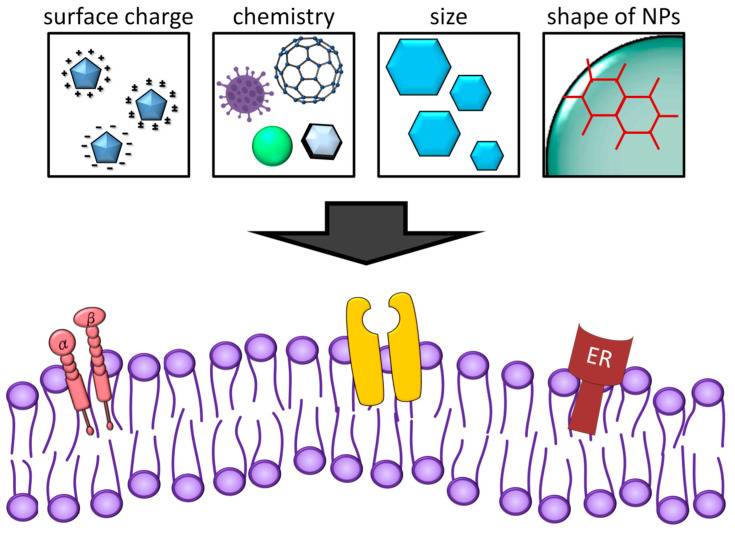
Factors affect cellular uptake of NPs. The NP uptake by cells is determined by their surface charge, chemistry, size, and shape.

Studies have been attempting to establish a correlation between NP material-specific parameters and cell uptake, in addition to cell physiological response and survival. The surface charge, chemistry, size, and shape of NP affect their cellular uptake (Figure 3). The surface charge-induced cytotoxicity of NP was the result of Coulombic interactions [75]. The negatively charged plasma membranes attracted positively charged NPs, to cause membrane destruction and proton pump effects [76,77]. For example, positively charged AuNPs maximally depolarize cell membranes, while other charged NPs have negligible effects [78]. When looking at truly insoluble particles, the effects of their physical presence and adsorption properties need to be considered. The physical presence of particles within macrophages affects the function of macrophages and may affect macrophages’ moving due to excess particles. The effects on macrophage mobility begin when about 6% of the cell volume is occupied by particles [79]. Interestingly, when NPs are involved, dyskinesias occur at a lower percentage of occupancy [80]. One possibility is that a key factor involved in the disruption of intracellular actin function is the total surface area of intracellular particles [81]. Impaired cell motility and function can lead to changes in necrosis. The frustrating phagocytotic process observed in macrophages probably provides more examples of asbestos fibers than can be up-taken. Studies by Poland et al. indicated that when macrophages encounter long carbon nanotubes, impaired cell motility and function of phagocytosis occur [82,83]. Adsorption of NP components into the extracellular space may also occur [84,85].

Ionizing radiation (IR) therapy for cancer patients may increase bone loss and the risk of fractures partially caused by the excessive and long-term release of reactive oxygen species (ROS). Treatment with cerium oxide NPs (CeONP) (nanoceria) provides an important multifunctional protective effect against IR-induced cell damage while increasing bone formation differentiation and subsequent new bone deposition [86]. Nanoceria is not cytotoxic to the human melanoma cell line (Mel1007) at doses up to 400 μg/mL and is dose-dependently internalized by cells [87]. Nanoceria reduces intracellular ROS levels that correlate with a dose-dependent decrease in angiogenic genes such as VEGF expression [87]. In general, it has been confirmed that nanotechnology-based procedures are more effective than conventional chemotherapy or radiotherapy, with minor side effects [88,89].

Another interesting area for NP application in medicine is neuro-degeneration. The neurodegenerative process begins with the aging of neurons. Aβ plaques, neurofibrillary tangles, Lewy bodies, and Pick’s body may appear in different parts of the brain and progress to Alzheimer’s disease, Parkinson’s disease, Huntington’s disease, amyotrophic lateral sclerosis, and other diseases [90,91,92]. No specific treatment for these diseases has been identified so far [93]. Although common treatments help to prevent the onset of the disease, the condition of patients with progressive neurodegenerative disease is usually not improved completely [94]. Nanoparticulated quercetin has been used in animal models of neurodegeneration, showing improvements over shorter periods. Indeed, intranasal administration of NPs involving quercetin, the construction of superparamagnetic NPs, and combination therapy with NPs such as quercetin and other drugs have been proposed for future research [93]. On the other hand, metal-containing NPs have been widely used in the diagnosis, monitoring, and treatment of CNS diseases [95]. However, studies have shown that inhaled NPs may settle in the olfactory mucosa and migrate to the central nervous system (CNS) [96,97], causing neurotoxicity that raises concerns regarding nanotoxicity of CNS induced by nano-medicinal materials. 

## 5. Nanotoxicology

In addition to many industrial and medical applications, there are certain toxicities linked to NPs and other nanomaterials [11,98,99]. The risk of nano-toxics is getting gaining more attention than before. For example, NPs may get penetrate into the dendritic cells of the airway wall. Dendritic cells are the primary antigen-presenting cells and play key roles in the orchestration of the innate and adaptive immune systems. Targeting dendritic cells by nanotechnology stands as a promising strategy for cancer immunotherapy. However, results suggest that the absorption of NPs can impair the function of these cells. The physicochemical properties of NPs influence their interactions with dendritic cells, thus altering the immune outcome of dendritic cells by changing their functions in the processes of maturation, homing, antigen processing, and antigen presentation [100]. Concerns are raised about whether if standard toxicological methods detect dysfunction of these cells, or are whether they are fairly minor. As nanotechnology evolves, people may be exposed to a wider variety of NPs, and such proposals will certainly be made. Determining how to respond to these recommendations is part of the nanotoxicology challenge. Nanotoxicology derives from the study of ultrafine particles. Could durable, long, and fine fibers deposited in the alveoli may function as new types of asbestos? Perhaps so: studies of several groups suggest that exposure to such materials should be controlled [75,101]. A further concern is that artificial NPs are incorporated into a wide range of products and can be unknowingly encountered by the general public. Thus, substantial attention has been paid to the potential risks of NPs. We need to have basic knowledge of these toxic effects to encounter them properly.

### 5.1. Damage to Cells Caused by Nanoparticles

Cytotoxicity is an important measure both for assessing the impact of nanomaterials on public health and for developing them for a variety of biomedical applications such as drug delivery and biosensing. The composition of NP probably plays a major role in the cytotoxic effect [102]. However, the genotoxicity detected is mainly due to the shape of the particles. The exact mechanism by which foam can affect toxicity is not yet well understood. However, the shape is likely to mediate the absorption and/or deposition of particles, at least in part. Cubic and octahedral CeO_2_NPs (nanocerias) were reported by Wang and collaborators [103] to cause higher cytotoxicity and lower antioxidant properties in HepG2 cells than rod-shaped CeO_2_NPs (nanocerias). Meanwhile, Forest and colleagues [104] demonstrated that rod-shaped CeO_2_NP (nanoceria) enhances significant and dose-dependent pro-inflammatory and cytotoxic effects on other in vitro cells (RAW264.7 cells) that were not present after exposure to cubic/octahedral NPs. Another in vivo study in mice reported that spherical NPs were the least toxic immediately after exposure [105].

Cytotoxicity caused by surface chemistry has many different origins that include surface-specific binding, non-specific protein binding, non-specific protein binding, and their denaturation (i.e., β-sheet formation) [106]; temperature/pH changes induced by membrane perturbations; and various direct-released toxins [107]. For example, an iron-magnetic NP coated with dendritic guanidine provided cell permeation similar to the human immunodeficiency virus-1 (HIV-TAT) transactivating peptide [108]. However, reports indicate that size-induced cytotoxicity is usually more complex due to the involvement of multiple material parameters. However, some reports suggest that dimensional effects are directly related to chemistry because of the high surface (chemical) activity associated with the specific surface area of small particles. The effects on surfactants are other concerns, but little is known about the adsorption of intracellular substances. Adsorption of extracellular material can increase the ability of NPs to cross cell membranes, which can remove the surface coating by lysosomes and expose bare NPs.

Although there is strong clinical evidence that shapes of NPs have a significant effect on cell fate (such as asbestosis) [109,110], the effect of NPs’ shape on cell response is not fully understood. Related studies included toxicity studies of carbon nanotubes that were found to induce significant cytotoxicity and even declared as new asbestos [111,112,113]. Direct plasma membrane penetration, endosomal loss, and chromosomal translocations were detected when the carbon nanotubes were fed to various cell lines. However, differences in the proportions, complex surface chemistry, and charge of the nanotube samples examined made it difficult to determine the definitive cause of the cytotoxicity. Therefore, it has been wondered whether a particular combination of NP form, chemical, filler, or all possible properties contributes to cytotoxicity. These graphene-structured nanotubes are single-layered or multi-layered and can carry contaminant metals derived from the manufacturing process to the surface. They are very strong and often many times wider. Carbon nanotubes have poor solubility in water, low biodegradability, and dispersity. In addition, toxicity problems are associated with the interaction between carbon nanotubes with biomolecules in tissues and organs. The effects may be involved in the proteome and genome [72].

Zinc oxide (ZnO) NPs at 10–40 nm cause genotoxicity via superoxide radical-induced oxidative stress resulting from mitochondrial damage in CHL/IU cells. The S9 mixture appears to contribute to a further increase in genotoxicity through the production of superoxide radicals by metabolic activation of ZnONPs [114]. There are no particular positive results regarding the effects of nanocerias on the early development of zebrafish [115], further evaluation of the nanotoxicity of nanocerias is still necessary. AgNPs have antimicrobial properties to attract interest and be used in medicine, biosensors and biotechnology, and household and healthcare-related products such as cosmetics. These beneficial effects are also offset by the higher chemical reactivity of these NPs due to their surface-area-to-volume ratio, leading to the increased formation of ROS within cells. However, AgNPs increase the formation of ROS. With increased human exposure to AgNPs, the risk of cytotoxicity and genotoxicity increases.

The effects of polyethylene glycol NPs (PLGA-PEG NP) on the physiological response in human cells are also investigated. PLGA is an FDA-approved biomedical material due to its biodegradability and biocompatibility. This is a very attractive candidate for drug delivery with controlled release, stealth, and targeting capabilities. The PLGA-PEGNP design is in a spherical or needle-like shape. The needle-shaped NPs were formed by directly stretching the synthesized spherical NPs to maintain the same volume, chemical properties, and charge. Needle-shaped NPs have been found to induce a series of physiological changes in cells when introduced into cells, ultimately causing significant cytotoxicity. 

NPs have the ability to be reactive due to their toxicological effects. One of the toxicities of NP is its ability to organize around protein concentrations that depend on the particle size, curvature, surface shape and properties, functional groups, and free energy. Due to this binding, some particles produce detrimental biological consequences through protein expansion, fibrillation, thiol cross-linking, and loss of enzyme activity. In addition, most NPs the are currently available have been designed according to their application and may not be natural. Thus, when the immune system detects NPs, the response may be tolerated and the NP is removed quietly without causing inflammation. On the other hand, the immune system may induce an activation. The responses are based on the size of the NPs, surface charge, and the hydrophobicity/hydrophilicity of the surface [116]. Generally, NPs with small size, hydrophilicity, and negative surface charge are tolerated [117]. NPs less than 4–6 nm are undetected after intravenous administration that undergoes renal clearance rapidly [116]. The NP becomes a target for various immune cells when the NP’s diameter increases. The interactions between NPs and the components of the immune system are fields to which we might pay much more attention in the future. Furthermore, NPs can invade organisms during ingestion or inhalation and migrate to various organs and tissues in the body. The potential adverse effects of NPs in different organs are listed in Table 2 and will be discussed in the following sections.

### 5.2. Effect of Nanoparticles in Different Organs

#### 5.2.1. Nanoparticles on Skin

The skin is the first place to contact most nanotechnologies. Therefore, it may be the earliest and prime target for nanotoxicity [145]. Furthermore, skin may be affected by many disorders that can be treated by topical applications of drugs on the action site. The application of NPs in dermatology and cosmetology represents a new field, closely related to the theme of risk assessment, as the potential and consequences of the penetration of these particles into living tissues have not been definitively determined [146]. With the advent of nanotechnologies, new efficient delivery systems have been developed [147]. The structural similarity between the nanosystem lipid matrix and the skin lipids allows the achievement of a transdermal effect [147]. Thus, some lipid-based nano-systems are focused on their use for topical application. In particular, dissolvable biocompatible nano-systems can control the release of pay-loaded drugs to potentially reduce side effects. In particular, the rationale for topical application of antioxidant molecules via lipid nanocarriers is available. Indeed, the structural similarity between the nano-system lipid matrix and the skin lipids allows the achievement of a transdermal effect [147]. Graphene oxide (GO) contains a large surface area, small size, and photothermal properties, which lend it potential to be used for drug delivery applications [148]. NanoGOs (GOns) are stable in water for over 6 months. A total of 55.5% of the mass of GOns dispersion permeate the skin in 6 h exposure but do not affect the human skin fibroblasts (HFF-1) morphology or viability. The small size and unique properties make GOns act as hapten, a substance that can combine with a specific antibody but lacks antigenicity of its own, and induce immune responses resulting in skin sensitization [149]. Different skin disorders cause elevated amounts of ROS including H2O2 in the epidermis [150]. Silica NPs showed high protein binding and induced cellular cytotoxicity via ROS [149] but no significant skin sensitization [149]. Polyethylene glycol (PEG) has been used as a linkers in drug delivery systems [151]. Despite the many benefits of PEGylation, the application and exposure of the PEG can induce toxicity such as an immune response. However, PEGylated-NPs did not lead to skin sensitization. PEGylated-AuNPs have been shown to be less toxic than AuNPs [152]. Thus, PEG coating may be used to reduce the cytotoxicity of nanomaterials [153].

#### 5.2.2. Nanoparticles in Brain

NPs have been shown to enter the sensory cells of the olfactory epithelium and are transported through the olfactory nerve to the olfactory lobe of the brain [153,154]. Metallic NPs pass or evade the blood-brain barrier to reach the CNS, and induce neurotoxicity [96]. The consequences are related to inflammation, oxidative stress, DNA and/or mitochondrial damage, and cell death. The potential mechanisms are mediated by microglial cell activation, inflammatory factor release, generation of reactive oxygen species, apoptosis, and/or autophagy in glial cells [95]. Glial cells, especially microglia and astrocytes, play an important role in the CNS. The dysfunction of microglia or astrocytes can damage the brain and contribute to the neurodegeneration seen in Alzheimer’s and Parkinson’s diseases. In addition, these processes increase the load on the CNS and accelerate the onset of neurodegenerative diseases. NPs may induce signaling pathways involved in the mechanism of glial neurotoxicity [155]. MNPs@SiO_2_(RITC) induces the activation of microglia by triggering excitotoxicity in neurons via D-serine secretion that highlights the importance of neurotoxicity mechanisms incurred by NP-induced microglial activation [119]. AgNPs did not induce oxidative stress by themselves in brain but Trolox potentiated oxidative stress in rats following exposure to AgNPs [126]. On the other hand, AuNPs induced dose-dependent cytotoxicity in human neural progenitor cells and rat brain [118].

In addition to aerosol, oral ingestion of NPs may also cause neurotoxicity. Studies in Wistar rats with oral IONP administration (100 mg/kg/day) induced neurotoxicity [156]. The ingestion of manufactured NPs in pregnant mothers may increase the probability of health concerns emerging in the next generation [120]. Carbon black NPs (CBNPs) induced oxidative injury. Dalia H Samak et al. conducted in novo studies to expose carbon black NPs to chicken embryos. The results indicated that mRNA gene transcripts of antioxidants, proinflammatory, and apoptotic pathways were altered in the brain of chicken embryos [120]. Exposure of CBNPs induces upregulating free radicals, especially contributing to gene expression regarding inflammation and subsequent cellular apoptosis at higher concentrations [120]. Zengjin Wang et al. developed a pregnant mouse model that demonstrated that oral exposures to ZrO_2_NPs during pregnancy are dangerous for fetal brain development, especially in early pregnancy [121]. These results suggest that NPs are able to cross multiple biological barriers and nanotoxicity to the fetus is highly dependent on stages of pregnancy and fetal development or the maturity of multiple biological barriers. Nanoparticle digestion may also cause neurotoxicity [122,123]. Silicon dioxide NPs (SiO_2_NPs) are widely used as additives in the food industry with controversial health risks. Silicon dioxide NPs induce neurobehavioral impairments by disrupting the microbiota–gut–brain axis [122]. SiO_2_NP-induced neurotoxic effects may occur through the distinctive gut–brain axis, showing no significant impact on either the gut–lung axis or gut–liver axis [122]. Silica nanoparticles may also promote α-synuclein aggregation and Parkinson’s disease pathology [123]. Other studies also indicate that titanium dioxide NPs via oral exposure lead to the adverse disturbance of gut microecology and locomotor activity in adult mice [124]. During pregnancy, exposure to titanium dioxide NPs causes intestinal dysbiosis and neurobehavioral impairments that are not significant postnatally but emerge in the adulthood of offspring [125]. Chen J et al. also indicted that zinc oxide NPs might induce neurobehavioral impairments via crosstalk of gut microbiota and serum/hippocampus metabolites [123].

#### 5.2.3. Nanoparticles in Eye

Nanoformulations have been widely explored as potential alternatives for traditional ophthalmic formulation approaches [157]. However, the study on the safety of nanomaterials in eyes is still in its early stages [158]. AgNPs can induce mitochondrial apoptosis in human retinal pigment epithelium cells [159]. Mesoporous silica NPs (MSiNPs) are one of the most well-studied inorganic NPs for the delivery of drugs [160] and MRI contrast agents [161]; however, exposure to Ag+ combined with MSiNPs at a safe dose induced more significant toxicity than the MSiNPs alone on the eye [158]. Severe corneal damage and dry eye were observed in rat models upon exposure to MSiNPs-Ag+ compared with MSiNPs [158]. The AgNPs-induced apoptosis in human retinal pigment epithelium cells occurs via the combination of cell cycle dysregulation and autophagy [159]. Even at a safe dose, Ag+ caused more significant toxicity than the MSiNPs alone [158]. Interestingly, apoptotic effects caused by AgNPs are significantly inhibited by T. gondii pre-infection by the suppression of NOX4-mediated ROS production [159]. Graphene oxide (GO) induced nanotoxicity during zebrafish embryogenesis. GO spontaneously infiltrated the chorion and entered the embryo via endocytosis to damage the mitochondria and primarily translocated to the eye and heart. GO promoted excessive ROS generation and induced oxidative stress to cause DNA damage and apoptosis [160,161,162].

#### 5.2.4. Nanoparticles in Lung

The respiratory system represents a unique target for the potential toxicity of NPs because it receives the entire cardiac output in addition to being an entry point for inhaled particles [163]. As we described previously, NPs may penetrate into the dendritic cells of the airway wall. NPs interfere with the normal functions of dendritic cells [100]. The widespread use of metal oxide NPs (MO_x_NPs) poses a risk of exposure that may lead to adverse health effects on humans. Studies have been conducted for toxicities of four different types of MO_x_ NPs (ZnO, SiO_2_, TiO_2_, and CeO_2_) in human bronchial epithelial cells [164]. High-dose (25 μg/mL) ZnONPs caused severe cytotoxicity with altered metabolism of amino acids, nucleotides, nucleosides, tricarboxylic acid cycle, lipids, inflammation/redox, and fatty acid oxidation, as well as the elevation of toxic and DNA damage related metabolites. Fewer metabolomic alterations were induced by low-dose (12.5 μg/mL) ZnONPs [164] and were less effective. On the other hand, the cells exposed to SiO_2_, TiO_2_, and CeO_2_ NPs induced less cytotoxicity, even at high doses with similar metabolomic alterations, although each type of NPs induced distinct changes of certain metabolites [164]. Potential metabolic mechanisms of MO_x_NP induced nanotoxicity in lung epithelial cells and demonstrated the sensitivity and feasibility of using metabolomic signatures to understand and predict nanotoxicity in vivo [164]. 

Consumer spray products of AgNPs emit risk [165] and cause lung disease burden [154]. AgNPs preferentially accumulated in organs such as the heart, lung, kidney in murine animals, and the circulation in the blood and fecal excretions showed higher AgNP contents in comparison with the AuNPs [166]. Toxicity mediated by small AgNP (≤20 nm) in lung cells is not only dependent on the level of particle internalization but also on the AgNP size and concentration, which may involve varying pathways as targets [128]. Pre-existing conditions modulate sensitivity to numerous xenobiotic exposures such as air pollution. AgNP exposure has been shown to disrupt the inflammatory resolution, specifically 14-hydroxy docosahexaenoic acid (14-HDHA), and 17-hydroxy docosahexaenoic acid (17-HDHA)-derived specialized pro-resolving lipid mediators (SPMs), in metabolic syndrome (MetS), contributing to exacerbated acute inflammatory responses [167]. Thus, identifying a potential mechanism responsible for enhanced susceptibility in MetS can be targeted for interventional therapeutic approaches. Phosphonate-based surface passivation is able to reduce MO_x_NP-induced pulmonary toxicity [140]. Suppression of PTPN6 exacerbates aluminum oxide NP-induced COPD-like lesions in mice through activation of the STAT pathway [141]. AgNPs are also shown to induce changes in gene expression with relevance to oxidative stress, apoptosis, and ion transport [166].

AuNPs are considered nontoxic upon acute exposure, at least when they are equal to or above 5 nm size. The redox-sensitive Nrf-2-mediated up-regulation of the cytoprotective role of Glyoxalase 1 (Glo1) has been shown crucially to protect cells from AuNPs-induced toxicity [168]. However, aggregated AuNPs have beenshown significant cellular uptake faster than single AuNPs at earlier exposure, although the uptake rate was similar at later time points [129]. In addition, single as well as aggregated AuNPs show similar translocation rates across the lung barrier model [129]. When cells are challenged with a pro-inflammatory/pro-oxidative insult, they become susceptible to the pro-apoptotic effect of AuNPs. The surviving cells undergo epigenetic changes associated with the onset of a partial epithelial-to-mesenchymal transition (EMT) process driven by the increase in dicarbonyl stress, consequent to Glo1 inactivation. Those observations raise the concerns of AuNPs’ adverse effect on lung epithelial cells.

#### 5.2.5. Nanoparticles in Liver

The liver is the site that passively accumulates well-dispersed NPs, making it an important test site for studying new nanomedicines and their clinical translations [142]. Many studies have reported the protective effect of CeO_2_NP on ROS overproduction and inflammatory processes. However, other studies have shown the important effects of these NPs on promoting oxidative stress by reducing cell viability through autophagy, apoptosis, and inflammation [143,169]. Studies by Zhao et al. indicated that mice treated daily with CeCl_3_ such as 2, 10, 20 mg/kg body weight for two months may cause ROS accumulation, lipid peroxidation, and reduced defense and lead to damaged hepatocytes [132]. The decrease in antioxidants may be due to decreased CeCl_3_-induced expression of stress-related genes such as SOD and CAT, causing cell apoptosis in the liver [170]. However, contradictory papers report cerium oxide-related liver toxicity or protection against oxidative stress and inflammation [142,170]. The levels of cerium oxide NPs (CeNPs) in blood and tissues were considerably low, but they were detected in feces in oral administration. These results suggest that CeNPs are not up-taken in the gastrointestinal system [171]. On the other hand, high concentrations of cerium were detected in all tissues after intravenous injection, especially in the liver and spleen [171]. Thus, intravenous injection but not oral administration of CeNPs may induce toxicities [171]. However, CeNPs are not detected in oral treatment and intravenous injection in urine.

AgNPs are used widely in nanomedicine and pharmaceutical products. Studies indicate that AgNPs may interact with organ structures of the liver, kidney, and testis to induce injury [122]. Evidence also indicates that smaller AgNPs pose a higher potential risk than the larger ones, which might be associated with their behavior, dissolution rate, bioavailability, and their probable variable toxicokinetics [134]. Intoxication of AgNPs in male rats upgraded liver function markers such as serum transaminases and alkaline phosphatase activities. Meanwhile, it decreased the serum levels of albumin and total proteins [133]. In addition, AgNP disturbed the oxidation homeostasis by the increased lipid peroxidation, the depleted glutathione, and the suppressed activity of superoxide dismutase and catalase [133]. AgNPs also induced an apoptotic reaction by the up-regulation of p53 and down-regulation of Bcl-2 expression, as examined in ref. [133]. Furthermore, AgNPs exhibited a marked elevation in liver DNA damage, hepatic effects after low-dose exposure to nanosilver, and early and long-lasting histological and ultrastructural alterations in rats. AgNPs interact with the anatomical structures of the liver in ways that could induce injury [172].

Although the distribution of AgNPs and AuNPs in animals was primarily deposited in the mononuclear phagocyte system (MPS) such as the liver and spleen, AuNPs seemed to be prominently stored in the liver [166]. In Sparus aurata liver organ culture, AuNPs induced more effects than Au+ to increase activities on catalase and glutathione reductase and to damage DNA and cellular membranes. The effects were dependent on the size, coating, and concentration of AuNPs [173]. Interestingly, AuNPs can incite a robust macrophage response in mice, and there are important species-specific differences in their biodistribution, excretion, and potential for toxicity [132]. A study conducted by Javiera Bahamonde et al. showed that mice exposed to AuNPs developed granulomas in the liver and transiently increased serum levels of the pro-inflammatory cytokine interleukin-18 but no such alterations were found in rats [132]. No fatalities were reported in mice but rats died within hours of AuNP administration. Differences in AuNP biodistribution and excretion were also detected between the two species. Rats have a higher relative accumulation of AuNPs in spleen and greater fecal excretion. Katarina Kozics et al. indicated that PEG-AuNPs had a relatively long blood circulation time in male Wistar rats [174]. Primarily, PEG-AuNPs accumulated in the liver and spleen and lasted for up to 28 days after administration [174]. AuNPs are considered to be relatively difficultly biodegraded, and to remain accumulated in organs/tissues for an extended period or permanently [175]. Therefore, the accumulation of PEG-AuNPs in the liver and spleen may cause late toxic effects [174].

#### 5.2.6. Nanoparticles in Kidney

The kidney is the major organ for blood filtration and waste elimination. It plays a crucial role in the transport and clearance of NPs in vivo. The interactions of NPs with different kidney compartments are determined by the size, shape, and surface chemistry of NPs [132]. Therefore, it is possible to modulate those parameters and precisely regulate the interactions between NPs and kidney compartments. The study by Ronghui Lei et al. indicate that nano-copper at 200 mg/kg/d for 5 d induced mitochondrial failure and enhanced ketogenesis, fatty acid β-oxidation, and glycolysis, resulting in nephrotoxicity and hepatotoxicity in rats [135]. The administration of CS-IONPs displayed the highest spleen iron accumulation. The ferrous sulfate (FeSO4)-treated group showed the highest kidney iron accumulation as compared with the other groups. The histopathological examination revealed that signs of toxicity were predominant for groups treated with Cit-IONPs or commercial FeSO_4_ [136]. 

AgNPs are used widely in food, cosmetics, and healthcare products. The effects of exposure to AgNPs on adults are well-documented. Long-term exposure to low-dose AgNPs enhanced the transformation of malignant cells into non-tumor BEAS-2B cells in vitro [176]. Long-term exposure to AgNP may damage the ultrastructural structure of the kidney by causing inflammation and the expression of cell survival factors [177]. In the long run, these changes may lead to the inhibition of beneficial apoptotic pathways and the promotion of renal necrotic cell death. Studies in animal models indicate that mothers being exposed to AgNP during the perinatal period caused chronic inflammation in their offspring which may persist into adulthood [178]. In addition, exposure to AgNPs altered the immune response of offspring to environmental stress. Progeny exposed to AgNP showed altered responses in splenocyte proliferation tests when challenged with lipopolysaccharide, concanavalin A, AgNP, or silver ions.

#### 5.2.7. Nanoparticles in Reproductive System

Previous studies have shown that many types of NPs can overcome certain biological barriers and have toxic effects on vital organs such as the brain, liver, and kidneys [179]. Only recently has attention been focused on the reproductive toxicity of nanomaterials. MO_x_NPs can pass the blood–testis barrier and accumulate in the testis. Although some MO_x_NPs have been shown to have protective effects on male germ cells, contradictory reports indicate that these NPs impair male fertility by interfering with spermatogenesis. Exposure to MO_x_NP can induce the overproduction of ROSs in both in vitro and in vivo studies. The consequences cause oxidative stress, a major molecular mechanism suggested to lead to germ cell toxicity. The latter causes subsequent damage to proteins, cell membranes, and DNA, which can ultimately lead to damage to the male reproductive system. MO_x_NPs can cross the blood–testis barrier and accumulate in the testis. MO_x_NPs may interfere with spermatogenesis to compromise male fertility. Exposure to MO_x_NPs may induce ROS overproduction, oxidative stress, and lead to germ-cell toxicity and, eventually, the impairment of the male reproductive system. MO_x_NPs can cross the blood–testis barrier and accumulate in the testis. MO_x_NPs may interfere with spermatogenesis to compromise male fertility. AgNPs could interact with the anatomical structures of the testis and induce injury [180]. The molecular mechanisms involved in NPs-induced toxicity in the reproductive system are not fully understood. However, studies indicate that NPs increase ROS production to induce oxidative stress and inflammation. Consequently, it causes damage at the molecular and genetic levels, cytotoxicity, and apoptosis [181]. Graphene oxide (GO) adhered to and enveloped the chorion of zebrafish embryos mainly via hydroxyl group interactions, blocked the pore canals of the chorionic membrane, and caused marked hypoxia and hatching delay. Furthermore, GO penetrated the chorion spontaneously and entered the embryo via endocytosis. It is primarily translocated to the eye, heart, and yolk sac regions via the circulatory system [169]. In these organs, GO induced excessive ROS generation, increased oxidative stress, and damaged mitochondria to induce DNA damage and apoptosis. GO also induced developmental malformation of the eye, cardiac/yolk sac edema, tail flexure, and heart rate reduction. In contrast to the common dose–effect relationships of NPs, the adverse effects of GO on heart rate and tail/spinal cord flexure increased and then decreased as the GO concentration increased [162].

#### 5.2.8. Nanoparticles in the Immune System

Over-production of ROS induced by NPs plays an important role to activate oxidative stress, inflammation, and DNA damage. Eventually, it causes structural alterations, DNA mutations, and cell death. A similar capacity of large aggregate patterns of TiO_2_NPs [182], Al_2_O_3_NPs [139], and Fe_2_O_3_NPs [183] increase oxidative stress. Several metallic NPs such as Ag, Fe_3_O_4_, CdSe/ZnS, and AuNPs have been shown to be bio-degradable [166]. However, the decomposition process for metallic NPs itself produces a high concentration of free radicals that may trigger an inflammatory immune response [184,185]. Chronic inflammation can be caused by the penetration of persistent non-biodegradable or micrometric large-size particles in the lungs. Examples of particle-induced granulomatosis include silicosis and asbestosis [116]. However, those examples may not fit the standard of NPs.

## 6. Conclusions Remark

This review provided an overview of NPs, their types, characterization, physicochemical properties, applications, and potential toxicities. Due to its small size, from a few nanometers to 500 nm, NP has a large surface area and is suitable for various applications. The synthesized forms can also be controlled. In addition to this, optical properties are also dominant in these dimensions, further increasing the importance of these materials in photocatalytic applications. Synthesis techniques help to control the specific morphology, size, and magnetic properties of NPs. In addition to aerosol and oral uptake of NPs from the environment, several types of medical applicative NPs have raised nanotoxicity concerns. Those up-taken NPs, especially metal NPs, may cause damage to different organs. They may also cause adverse in the fetus or offspring at late-stage development in adults via pregnant mothers. Therefore, although NPs are useful in many applications, there are still some health issues due to uncontrolled use and emissions to the natural environment that should be considered to make NP use more convenient and environmentally friendly. 

## Figures and Tables

**Table 2:** Nanoparticle-induced toxicities in different organs.

Organ	Formation	Nanotoxicities	References
Brain	MNPs@SiO_2_(RITC)	Silica-coated magnetic NPs activate microglia and induce neurotoxic D-serine secretion	[118,119]
	IONP	Neurotoxic potential of iron oxide NPs in Wistar Rats	[118]
	Carbon black nanoparticles (CBNPs)	Exposure of carbon black NPs to chicken embryos	[120]
	ZrO_2_ NP	Breakthrough of ZrO_2_ NPs into fetal brains depends on developmental stage of maternal placental barrier and fetal blood–brain barrier	[121]
	Silicon dioxide NPs	Silicon dioxide NPs induced neurobehavioral impairments by disrupting microbiota–gut–brain axis.	[122,123]
	zinc oxide NPs	Crosstalk of gut microbiota and serum/hippocampus metabolites in neurobehavioral impairments induced by zinc oxide NPs.	[122,123]
	Silica NPs	Silica NPs promote α-Synuclein aggregation and Parkinson’s disease pathology.	[122,123]
	Titanium dioxide nanoparticles	Titanium dioxide NPs via oral exposure leads to locomotor activity in adult mice.	[124]
	Titanium dioxide nanoparticles	Titanium dioxide NPs exposure during pregnancy causes neurobehavioral impairments that emerge in offspring adulthood.	[125]
	AgNPs	Trolox potentiated oxidative stress in rats following exposure to AgNPs. However, AgNPs did not induce oxidative stress by themselves in brain.	[126]
	AuNPs	AuNPs induced dose-dependent cytotoxicity in human neural progenitor cells and rat brain.	[127,128]
Lung	MO_x_ NPs	Toxicities of four different types of MO_x_ NPs (ZnO, SiO_2_, TiO_2_, and CeO_2_) in human bronchial epithelial cells.	[127]
	AgNPs	The low dose of AgNPs induced early and long-lasting histological and ultrastructural alterations in rats.	[127]
	AgNP	Toxicity mediated by small AgNP (≤20 nm) in lung cells is not only dependent on the level of particle internalization, but also on AgNP size and concentration, which may involve varying pathways as targets	[128]
	AgNP	Low-dose AgNP exposure induced histological and ultrastructural alterations in rats’ lungs.	[127]
	AuNPs	Single as well as aggregated AuNPs show similar translocation rates across the lung barrier model.	[129]
	ZnONPs	High-dose (25 μg/mL) ZnO NPs caused severe cytotoxicity.	[127]
Heart	CdSe/ZnS Quantum dots	Quantum dots might build up in the heart and induce some biochemical indicators. The consequence alternated and caused oxidative damage and cardiotoxicity.	[130]
Liver	CeO_2_NP	Iron oxide NPs aggravate hepatic steatosis and liver injury.	[130]
	Iron oxide NP	Hepatotoxicity of graphene oxide in Wistar rats.	[131]
	Graphene oxide	AuNPs induced species-specific differences in their biodistribution, excretion, and potential for toxicity.	[132]
	AuNP	AuNPs caused granulomas to develop in the mice’s livers and transiently increased serum levels of the pro-inflammatory cytokine interleukin-18.	[133]
	AgNP	AgNPs intoxicated liver by elevating the liver function markers and decreased serum levels of albumin and total proteins. It also disturbed oxidation homeostasis and induced apoptotic reaction.	[127]
	AgNP	AgNPs exhibited a marked elevation in liver DNA damage.	[134]
	AgNP	The low dose of AgNP induced hepatotoxicity showing early and long-lasting histological and ultrastructural alterations in male rats.	[127,134]
	AgNP	In vivo study of silver nanomaterials’ toxicity concerning size.	[134]
Kidney	Nano-copper particle	The nano-sized copper particle induced hepatotoxicity and nephrotoxicity in rats.	[135]
	IONP	Surface modifications affect iron oxide NP biodistribution in rats.	[136]
	AgNP	Single silver nanoparticle instillation induced early and persisting moderate cortical damage in rat kidneys.	[134]
	AgNP	AgNPs could interact with the anatomical structures of the kidney to induce injury.	[134]
Reproductive system	Metal oxide NPs (MONPs)	MONPs may induce ROS overproduction, oxidative stress, and lead to germ cells’ toxicity. Eventual, consequence of the impairment of the male reproductive system.	[137]
	AgNPs	AgNPs could interact with the anatomical structures of testis and induce injury.	[134]
Blood	AuNPs	Trigger platelet aggregation	[138]
	TiO_2_NPs Al_2_O_3_NPs, Fe_2_O_3_NPs	Aggregated NPs increase oxidative stress and immune response.	[139,140,141]
	Ag, Fe_3_O_4_, CdSe/ZnS, AuNPs	Several metallic NPs such as Ag, Fe_3_O_4_, CdSe/ZnS, and AuNPs have been shown to be bio-degradable and produces a high concentration of free radicals that may trigger an inflammatory immune response.	[142,143,144]

## Data Availability

No new data were created or analyzed in this study. Data sharing is not applicable to this article.
